# The coexistence of arbuscular mycorrhizal fungi and dark septate endophytes synergistically enhanced the cadmium tolerance of maize

**DOI:** 10.3389/fpls.2024.1349202

**Published:** 2024-05-24

**Authors:** Zhaodi Wang, Lei Wang, Xinran Liang, Guangqun Zhang, Zuran Li, Zhixin Yang, Fangdong Zhan

**Affiliations:** ^1^ College of Resources and Environment, Yunnan Agricultural University, Kunming, Yunnan, China; ^2^ College of Landscape and Horticulture, Yunnan Agricultural University, Kunming, Yunnan, China

**Keywords:** endophytic fungi, cadmium stress, glutathione metabolism, endogenous hormone, synergistic effect

## Abstract

**Introduction:**

Arbuscular mycorrhizal fungi (AMF) and dark septate endophytic fungi (DSEs) generally coexist in the roots of plants. However, our understanding of the effects of their coexistence on plant growth and stress resistance is limited.

**Methods:**

In the present study, the effects of single and dual inoculation of AMF and DSE on the growth, photosynthetic physiology, glutathione (GSH) metabolism, endogenous hormones, and cadmium (Cd) content of maize under 25 mg•kg^-1^ Cd stress were investigated.

**Results:**

Compared with that after the non-inoculation treatment, AMF+DSE co-inoculation significantly increased the photosynthetic rate (Pn) of maize leaves; promoted root GSH metabolism; increased the root GSH concentration and activity of γ-glutamyl cysteine synthase (γ-GCS), ATP sulfatase (ATPS) and sulfite reductase (SIR) by 215%, 117%, 50%, and 36%, respectively; and increased the concentration of endogenous hormones in roots, with increases in zeatin (ZR), indole-3 acetic acid (IAA), and abscisic acid (ABA) by 81%, 209%, and 72%, respectively. AMF inoculation, DSE inoculation and AMF+DSE co-inoculation significantly increased maize biomass, and single inoculation with AMF or DSE increased the Cd concentration in roots by 104% or 120%, respectively. Moreover, significant or highly significant positive correlations were observed between the contents of ZR, IAA, and ABA and the activities of γ-GCS, ATPS, and SIR and the glutathione (GSH) content. There were significant or highly significant positive interactions between AMF and DSE on the Pn of leaves, root GSH metabolism, and endogenous hormone contents according to two-way analysis of variance. Therefore, the coexistence of AMF and DSE synergistically enhanced the Cd tolerance of maize.

## 1 Introduction

Mining, metal smelting, chemical production, pesticide application, fertilizer application, and other production activities have aggravated soil heavy metal pollution in industrial and mining areas (Veltman et al., 2008). For example, soil cadmium (Cd) pollution is a serious problem in China according to the National Soil Pollution Survey Bulletin (Chen et al., 2019). Plants absorb and accumulate Cd in their organs, which affects their physiological and metabolic processes of plants, such as photosynthesis (Dias et al., 2013), glutathione metabolism, and endogenous hormones (Wang et al., 2012), and inhibits their plants.

Arbuscular mycorrhizal fungi (AMF) and dark septate endophytes (DSE) are widely distributed in natural ecosystems and colonize plant roots in Cd-polluted soils (Liang et al., 2007). The AMF forms a mycorrhizal association with approximately 80% of plants in terrestrial ecosystems (Aseel et al., 2019). and are beneficial to the growth of host plants via increasing nutrient absorption and improving host photosynthesis (Farzaneh et al., 2011). Under Cd stress, AMF increases the net photosynthetic rates of host plants (Zhang et al., 2017), promotes GSH metabolism in crops, alleviates the hormone imbalances caused by Cd stress (Zhan et al., 2017) and reduces the toxic effects of Cd stress on plants (AbdaAllah et al., 2015). AMF colonization in rice roots helped to improve rice tolerance to metals by stabilizing the Cd content in the rhizosphere and the Zn content in the root tissues where the fungal hyphal ramification was prominent (Janeeshma and Puthur, 2022). AMF promoted Cd binding to plant roots, inhibited Cd transport to leaves, and reduced Cd concentrations in leaves (Gu et al., 2017).

DSEs can also increase the nutrient acquisition of host plants, promote plant growth (Wang et al., 2016), resist Cd toxicity, and enhance metabolic activity against Cd stress (Selosse et al., 2004; Gadd, 2007). DSEs enhance plant adaptability to Cd by regulating GSH metabolism (Pan et al., 2016) and plant hormones concentrations in host plants (Singh et al., 2015). Additionally, the DSE promoted the accumulation of Cd in plant roots and reduced the migration of Cd from roots to leaves, which reduced the Cd content in leaves (Zhang et al., 2013).

Notably, AMF and DSEs colonize plant roots simultaneously (He et al., 2017). Some studies have investigated the effects of their co-inoculation on plant growth and physiology (Khaekhum et al., 2021). Single inoculation or co-inoculation of AMF and DSEs increased the maize biomass, mineral nutrient content, and growth under Cd stress (He et al., 2019). A single inoculation of AMF and co-inoculation of AMF+DSE significantly increased the biomass of maize, promoted Cd retention in roots, and reduced the Cd content in shoots (He et al., 2020). Thus, AMF and DSEs coexist in plant roots and promote plant resistance under adverse environmental stress (Bi et al., 2019). However, the physiological mechanism by which the co-inoculation of AMF and DSE enhances the Cd tolerance of plants is still unclear.

To determine whether AMF and DSE coexist in plant roots under Cd stress, the present study investigated the effects of single and double inoculation with AMF and DSE on maize growth, photosynthesis physiology, root GSH metabolism physiology, root endogenous hormone contents, and plant Cd content. The relationships between the growth physiology and Cd content in maize were analyzed. We hypothesized that the coexistence of AMF and DSEs in roots would synergistically enhance the Cd tolerance of the host plant by improving the photosynthetic physiology in leaves, endogenous hormone contents and GSH metabolism in roots.

## 2 Materials and methods

### 2.1 Materials tested

The soil type was mountain red soil, which was naturally dried indoors and then passed through a 2 mm sieve. The chemical properties of the soil were as follows: pH, 6.35; organic matter content, 13.87 g·kg^-1^; total nitrogen, phosphorus, and potassium contents, 0.112, 0.32, and 1.98 g·kg^-1^, respectively; alkali-hydrolyzable nitrogen, available phosphorus and available potassium contents, 26.18, 1.15 and 33.74 mg·kg^-1^, and soil Cd content, 0.275 mg·kg^-1^. Moreover, the sands were also screened through a 2-mm sieve after natural air drying. The soil and sand were fully mixed at a ratio of 3:1 (w/w) of sand to soil. Then, a mixture of sand and soil was added with CdCl_2_·2.5 H_2_O solution to achieve a Cd stress level of 25 mg·kg^-1^, which was balanced for 2 weeks before use in the pot experiment.

The AMF used in the experiment was *Funneliformis mosseae* (*F. mosseae*) BGC YN05 1511C0001BGCAM0013, which was provided by the Institute of Plant Nutrition and Resources, Beijing Academy of Agricultural and Forestry Sciences. The AMF inoculants (including spores, mycelia, sand mixtures and infected root segments, etc.) were obtained by the propagation of potted maize in the laboratory, and there were 30 spores per gram of inoculant. The DSE strain (*Exophiala pisciphila* (*E. pisciphila*) ACCC32496) was isolated from the root of *Arundinella bengalensis* (Spreng.) Druce naturally grows in the Huize lead-zinc mine area, in Yunnan Province. It was preserved at a low temperatures (4°C) in the laboratory. The DSE fungus was inoculated on the potato dextrose agar (PDA) medium, and cultured at 28°C for 2 weeks to activate the strain.

The trial maize (*Zea mays* L.) was Huidan No. 4. After surface disinfection with 10% hypochlorous acid (soaked for 2 min) and 75% ethanol (soaked for 1 min), the maize seeds were placed for 3 days in a constant temperature incubator at 28°C for germination. After the seeds germinated and sprouted, the unpolluted and healthy seedlings were selected for subsequent use.

### 2.2 Pot experiment

The establishment of the maize-DSE symbiont: A cylindrical glass bottle with 20 cm (height) and 6.5 cm (diameter) in height was used, in which 16.5 g of perlite and 20 mL of Hoagland nutrient solution were added. The device was autoclaved at 121°C for 30 min. For the DSE-inoculated treatment, 15 pieces of DSE colonies (2 mm diameter) from a potato dextrose agar (PDA) culture were added to each of the eight glass bottles. After mixing well with perlite, 2 maize seeds were added, covered with sterile sand to a thickness of 1 cm and sealed with sterile polyvinyl alcohol film (PVA). During the growth of maize plants, maize roots grew and attached to DSE colonies, therefore the DSE mycelium infested with maize roots. For the no-DSE-inoculated treatment, 15 autoclaved fungal disks were used, and the maize seedlings were placed in glass bottles. The maize seedlings were cultivated for 14 days at 25°C under light conditions of 1000–8000 lux for 10 hours per day. Some maize plants were sampled and examined under a the microscope to confirm the successful DSE colonization of DSE in the roots.

For the indoor pot experiments, plastic pots with a height of 20 cm, an upper diameter of 25 cm, and a bottom diameter of 21 cm were sterilized with 75% ethanol. Then, 5 kg of sterilized soil-sand mixture was added, and two maize seedlings were planted in each pot. The pot experiment was performed in a completely randomized design with the following variables: one Cd concentration (25 mg·kg^-1^), 4 treatments and 6 replicates in each treatment, for a total of 24 pots. The four treatments were as follows: the control treatment (CK) consisted of no AMF or DSE inoculation, AMF inoculation, DSE inoculation, and AMF+DSE co-inoculation. In the CK treatment, maize plants were not inoculated with DSE or AMF. For the AMF inoculation treatment, the maize plants without DSE colonization were inoculated with 50 g of AMF inoculants (approximately 1500 spores per pot) without DSE. For the DSE inoculation treatment, the maize plants were cultivated in glass bottles with DSE colonization, and an equal amount of sterilized AMF inoculants was added. For the AMF+DSE co-inoculation treatment, the maize plants with DSE colonization were added with 50 g of AMF. Each treatment had 6 pots, and one maize plant was planted per pot.

After planting, 150 mL of 50% Hoagland nutrient solution was added to the maize plants every 3 days. The soil moisture was kept constant at 55% by weighing and replenishing the water.

### 2.3 Determination of maize biomass and photosynthesis in maize leaves

At approximately 12:00 p.m., the photosynthetic rate (Pn), transpiration rate (Tr), stomatal conductance (Gs), and intercellular CO_2_ concentration (Ci) in the maize leaves were measured using an LCA-4 photosynthetic tester (Analytical Development Co., Ltd., ADC, Hoddesdon, England). The maize was harvested after photosynthetic index measurement, and other indices were measured. For each treatment, the photosynthetic physiological parameters were collected from the fourth leaf from the top of maize plant.

After being planted for sixty days, the maize plants were harvested and divided into leaves and roots. The plants were washed with distilled water, dried in a 105°C oven (Model 101, Beijing Yongguangming Instrument Co., Ltd, Beijing, China) for 0.5 h and then dried in a 75°C oven for 48 h to a constant weight, after which their dry biomass was weighed.

### 2.4 Determination of AMF and DSE colonization in roots

The AMF spores in the maize rhizosphere soils were separated via wet sieve decantation-sucrose centrifugation, and the number of spores was observed and calculated under a microscope (Daniels and Skipper, 1982).

Some fresh roots were randomly sampled, washed with water, and then cut into fragments approximately 1 cm in length. First, these root fragments were placed into a centrifuge tube, with 10% KOH solution, incubated in a water bath at 90°C for 90 min, and then washed with water. Second, they were acidified and dissociated by lactic acid acidification at room temperature for 5 min, washed with water. Third, they were stained with 0.05% Tepan blue staining solution, at 90°C for 30 min and washed with water. Finally, the plants were decolorized with a decolorization solution at room temperature for 1 d. Ten root fragments were randomly selected 10 root segments were placed on slides, and sealed with 50% glycerol (Chen et al., 2021), and observed under a microscope. The colonization rates of AMF and DSE were calculated using the intersection method (McGonigle et al., 1990).

### 2.5 Determination of glutathione metabolism in maize roots

For sample preparation, fresh roots (0.1 g) were weighed 0.9% saline solution (2 mL) was added, and the roots were ground into a homogenate using a high-throughput tissue grinder (Scientitz-48, Shanghai Shengke Instrument Equipment Co., Ltd., Shanghai, China). These roots were transferred to a 2-mL centrifuge tube and placed in a high-speed freezing centrifuge (HC-3018R, Anhui Ustc Zonkia Scientific Instrument Co., Ltd., Anhui, China). Then, the samples were centrifuged at 12000 ×g for 30 min, and the supernatant was collected for analysis.

The glutathione (GSH) content was determined using the method described in the GSH test kit produced by Nanjing Jiancheng Bioengineering Institute, according to previous methods (Wang et al., 2023). The supernatant obtained by centrifugation was used for the determination of the GSH content by measuring the absorbance at 405 nm.

Glutathione reductase (GR) and γ-glutamylcysteine synthetase (γ-GCS) activities were determined using the methods described in the GR and γ-GCS test kits produced by Nanjing Jiancheng Bioengineering Institute, according to previous methods (Zhan et al., 2017). One unit of GR activity is equal to the oxidation of 1 nmol of NADPH per minute. The γ-GCS activity was defined as the amount of enzyme required to consume 1 μmol of NADH per minute.

ATPS (ATP sulfatase), OAS-TL (O-acetyl serine (thiol) lyase), and SIR (sulfite reductase) activities were measured using the plant ATPS kit, OAS-TL kit, and SIR kit, respectively, produced by Shanghai Jingkang Bioengineering Co., Ltd. Enzyme-linked immunosorbent assay (ELISA) with double antibodies was performed using kits. The specimen, standard, and HRP-labeled detection antibody were added to the coating micropores, which were coated with plant ATPS, OAS-TL, and SIR capture antibodies in advance, incubated, and thoroughly washed. The substrate 3, 3’, 5,5’-tetramethylbenzidine (TMB) was used for color development. TMB is converted to blue under the catalysis of peroxidase and finally to yellow under the action of acid. The depth of color positively correlated with the plant ATPS, OAS-TL, and SIR in the samples. The absorbance (OD) was measured at 450 nm and the concentrations of ATPS, OAS-TL, and SIR in the samples were calculated from the standard curve.

### 2.6 Determination of endogenous hormones in maize roots

Maize roots (2.0 g) were sampled and cleaned thoroughly with water, put into a mortar with 5 mL of 80% ice methanol and ground to a homogenate for 10 min. The homogenate was transferred to a conical flask (100 mL) 20 mL of 80% ice methanol was added, and the mixture was shaken well and incubated at 0°C for 40 h. Then, the homogenate was filtered and concentrated to 10 mL under reduced pressure. The concentrations of zeatin (ZR), gibberellic acid (GA), indole acetic acid (IAA), and abscisic acid (ABA) in the homogenate were measured by high-performance liquid chromatography (HPLC, Thermo Fisher Scientific Ultimate 3000, Shanghai Lijing Scientific Instrument Co., Ltd.) A Diamonsil C18 column (4.6 mm×250 mm, 5 μm) was used as the chromatographic column, and methanol and water formed the mobile phase. The flow rate and temperature were set at 1.0 mL·min^-1^ and 25°C, respectively, and the injection volume was 10 μL. The detection wavelengths of ZR, GA, IAA, and ABA was at 254 nm (Wei et al., 2013). The retention times of ZR, GA, IAA, and ABA in the standard samples were 5.645, 4.366, 6.514, and 11.942 min, respectively.

### 2.7 Determination of Cd content in maize

The dried shoot and root samples (0.1 g) were weighed, ground into powder and digested using the nitric acid-perchloric acid (4:1) via the wet-digestion method. The digestion mixture was transferred to a volumetric flask and diluted to 50 mL with distilled water. The Cd concentration in solution was determined using flame atomic absorption spectrophotometry (TAS-990, Beijing Puxi Instrument Factory, Beijing, China). The appropriate quality control was determined using CdCl_2_ as the standard solution. The Cd content (mg·kg^-1^) the plants was calculated according to the formula by Bao (Bao, 2000). The Cd accumulation in maize shoots or roots was equal to the Cd content in shoots or roots multiplied by their biomass.

### 2.8 Data analysis

The data are presented as the average of 6 replicates with the average standard deviation. The data figures were drawn using Origin Pro 9.0 and statistically analyzed using SPSS 25.0. The significance of the difference at 0.05 was detected using least significant difference (LSD). Two-way Analysis of Variance (ANOVA) was used to evaluate the effects of AMF and DSE inoculation on maize growth physiology and the interaction between AMF and DSE. The correlation coefficients between endogenous hormone content in maize roots and GSH metabolism and photosynthesis physiology in shoots of maize were analyzed by Pearson correlation.

## 3 Results

### 3.1 Colonization of maize roots by AMF and DSEs

In the single AMF or DSE inoculation and AMF+DSE co-inoculation treatments, AMF or DSE structures such as hyphae and spores were observed in the maize roots. However, these compounds were not detected in the roots of the maize plants in the non-inoculation treatment (CK).The number of AMF spores in the AMF inoculation treatment was greater than that in the AMF+DSE co-inoculation treatment. There was no significant difference in the AMF colonization rate between AMF inoculation and AMF+DSE co-inoculation treatments, and there was no significant difference in the DSE colonization rate between DSE inoculation and AMF+DSE co-inoculation treatments. These results indicated that AMF and DSE successfully established a symbiotic relationship with maize (**Table 1**).

**Table 1:** Spore number of AMF and root colonization of AMF and DSE.

Treatment	Spore number of AMF (n/g)	AMF colonization rate (%)	DSE colonization rate (%)
CK	—	—	—
AMF	8.6 ± 0.44 a	49.82 ± 2.58 a	—
DSE	—	—	28.51 ± 4.85 a
AMF+DSE	7.1 ± 0.65 b	53.15 ± 2.21 a	25.48 ± 2.29 a

CK, the control of non-inoculation; AMF, *Funneliformis mosseae* inoculation; DSE, *Exophiala pisciphila* inoculation; AMF+DSE, co-inoculation of *F. mosseae* and *E. pisciphila*. “—” indicates the index of the material was not detected. The different lowercase letters in a column indicate significant differences among treatments at *p*<0.05 level.

### 3.2 Effects of AMF and DSEs on maize growth

**Figure 1:**
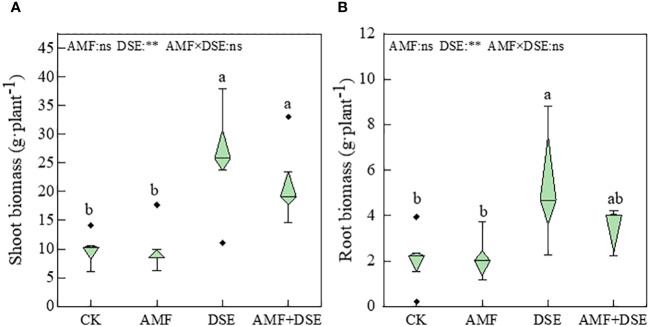
Effects of AMF and DSE on maize growth under Cd stress: **(A)** Shoot biomass; **(B)** Root biomass. The different lowercase letters and the same lowercase letters indicate significant and non-significant differences among treatments (*p* < 0.05), respectively. CK, the control of non-inoculation; AMF, *F. mosseae* inoculation; DSE, *E. pisciphila* inoculation; AMF+DSE, co-inoculation of *F. mosseae* and *E. pisciphila*. “ns”, “*” and “**” means no significant, and *p*< 0.01 according to two-way ANOVA, respectively.

Compared with the CK treatment, the DSE inoculation and AMF+DSE co-inoculation treatments significantly increased the biomass of maize leaves by 26% and 15%, respectively. The DSE- inoculation treatment also significantly increased the root biomass. According to the two-way ANOVA,DSE significantly increased maize biomass, but there was no interaction between AMF and DSE on maize biomass (**Figure 1**). These results indicated that the single inoculation with DSE and co-inoculation with AMF+DSE promoted maize growth.

### 3.3 Effects of AMF and DSEs on the photosynthesis of maize leaves

**Figure 2:**
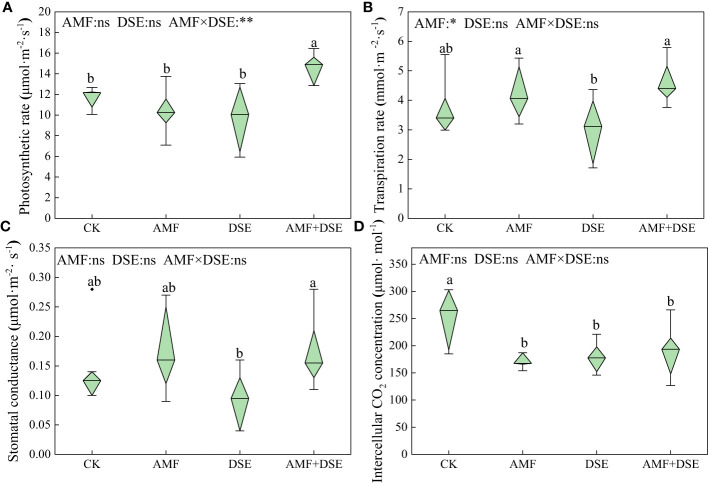
Effects of AMF and DSE on photosynthesis of maize leaves under Cd stress: **(A)** Photosynthetic rate; **(B)** Transpiration rate; **(C)** Stomatal conductance; **(D)** Intercellular CO_2_ concentration. The and the same lowercase letters different lowercase letters indicate significant and non-significant differences among treatments (*p* < 0.05), respectively. CK, the control of non-inoculation; AMF, *F. mosseae* inoculation; DSE, *E. pisciphila* inoculation; AMF+DSE, co-inoculation of *F. mosseae* and *E. pisciphila*. “ns”, “*” and “**” means no significant, and *p* < 0.01 according to two-way ANOVA, respectively.

AMF+DSE co-inoculation enhanced the photosynthetic physiology of maize leaves. Compared with that after the CK treatment, AMF+DSE co-inoculation significantly increased the photosynthetic rate of leaves by 24.9%. AMF inoculation, DSE inoculation, and AMF+DSE co-inoculation significantly reduced the intercellular CO_2_ concentration by 25.3%, 29.1%, and 24.5%, respectively. According to the two-way ANOVA,AMF significantly increased the transpiration rate of maize leaves. There was a very significant interaction effect between AMF and DSE on the photosynthetic rate (**Figure 2**).

### 3.4 Effects of AMF and DSEs on glutathione metabolism in maize roots

**Figure 3:**
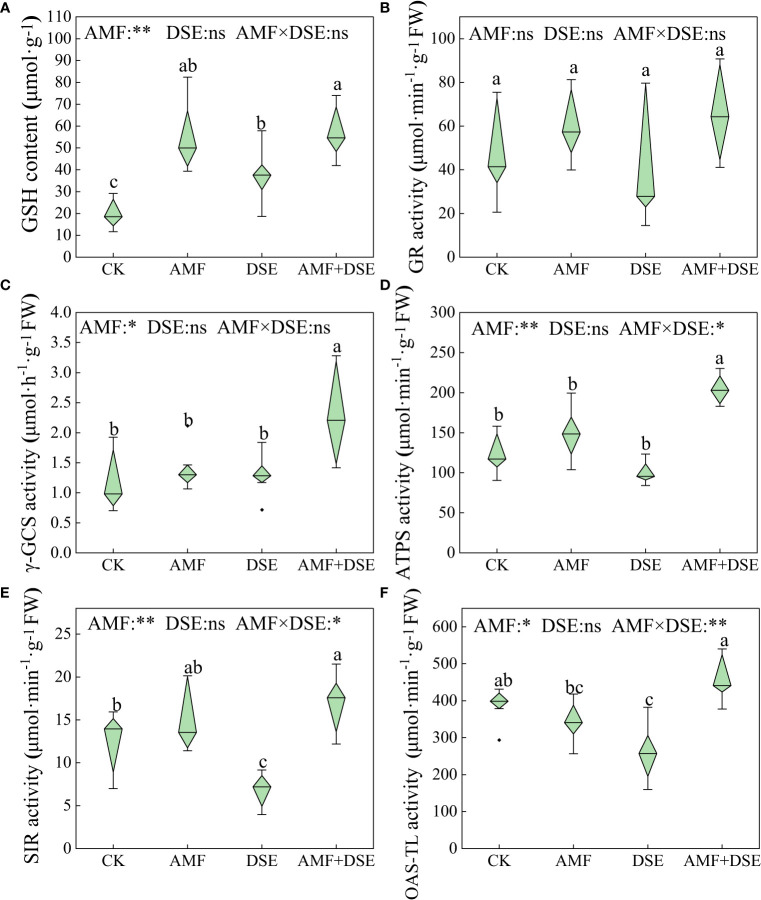
Effects of AMF and DSE on glutathione metabolism of maize roots under Cd stress: **(A)** GSH content; **(B)** GR activity; **(C)** γ-GCS activity; **(D)** ATPS activity; **(E)** SIR activity; **(F)** OAS-TL activity. The different lowercase letters and the same lowercase letters indicate significant and non-significant differences among treatments (*p* < 0.05), respectively. CK, the control of non-inoculation; AMF, *F. mosseae* inoculation; DSE, *E. pisciphila* inoculation; AMF+DSE, co-inoculation of *F. mosseae* and *E. pisciphila*. “ns”, “*” and “**” means no significant, and *p*< 0.01 according to two-way ANOVA, respectively.

AMF inoculation and AMF+DSE co-inoculation enhanced GSH metabolism in maize roots. Compared with those in the CK group, the activities of γ-GCS and ATPS in the AMF+DSE co-inoculation group were significantly increased by 117.3% and 50.0%, respectively. According to the two-way ANOVA, AMF had significant effects on the activities of ATPS, SIR, γ-GCS and OAS-TL, and the GSH content in maize roots. There was a substantial interaction effect between AMF and DSE on the activities of OAS-TL, ATPS, and SIR (**Figure 3**).

### 3.5 Effects of AMF and DSEs on the endogenous hormone contents in maize roots

**Figure 4:**
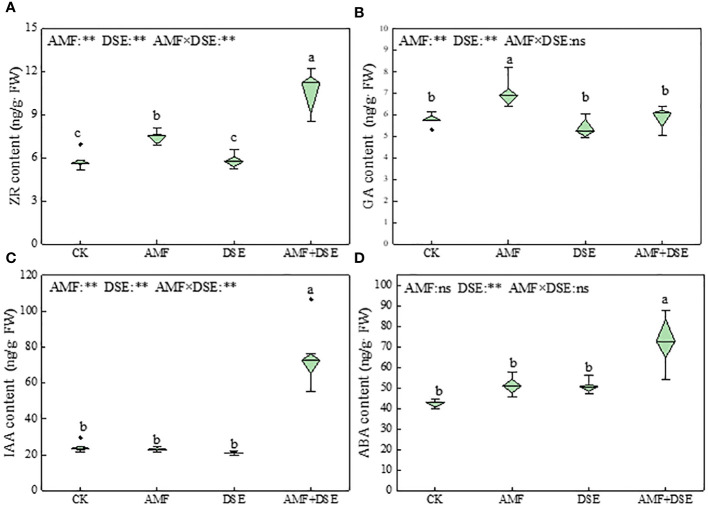
Effects of AMF and DSE on endogenous hormone content in maize roots under Cd stress: **(A)** ZR content; **(B)** GA content; **(C)** IAA content; **(D)** ABA content. The different lowercase letters and the same lowercase letters indicate significant and non-significant differences among treatments (*p* < 0.05), respectively. CK, the control of non-inoculation; AMF, *F. mosseae* inoculation; DSE, *E. pisciphila* inoculation; AMF+DSE, co-inoculation of *F. mosseae* and *E. pisciphila*. “ns”, “*” and “**” means no significant, and *p* < 0.01 according to two-way ANOVA, respectively.

AMF inoculation and AMF+DSE co-inoculation promoted the production of endogenous hormones in maize roots. Compared with those following the CK treatment, AMF+DSE co-inoculation significantly increased the contents of ZR, IAA, and ABA by 81%, 209%, and 72%, respectively. AMF inoculation significantly increased the contents of ZR and GA by 27% and 21%, respectively. According to the two-way ANOVA results showed that AMF and DSE had significant effects on the contents of ZR, GA, IAA, and ABA. There was a very significant interaction effect between AMF and DSE on the contents of ZR and IAA (**Figure 4**).

### 3.6 Effects of AMF and DSEs on Cd accumulation in maize

**Figure 5:**
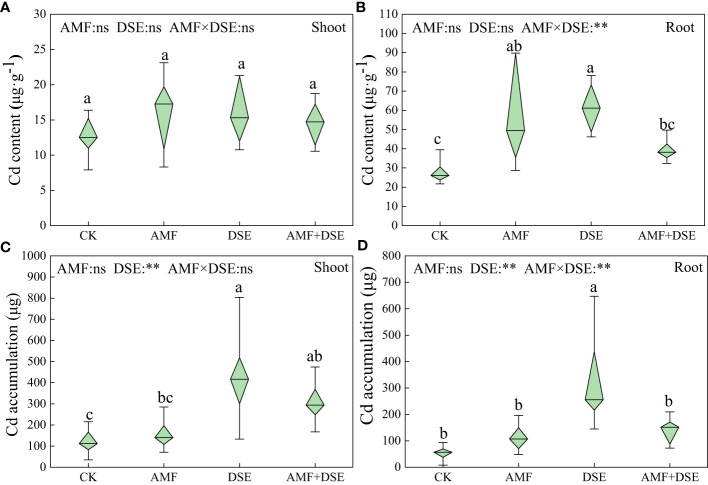
Effects of AMF and DSE on accumulation characteristics in maize under Cd stress: **(A)** Cd content in maize shoot; **(B)** Cd content in maize root; **(C)** Cd accumulation in maize shoot; **(D)** Cd accumulation in maize root. The and the same lowercase letters different lowercase letters indicate significant and non-significant differences among treatments (*p* < 0.05), respectively. CK, the control of non-inoculation; AMF, *F. mosseae* inoculation; DSE, *E. a pisciphila* inoculation; AMF+DSE, co-inoculation of *F. mosseae* and *E. pisciphila*. “ns”, “*” and “**” means no significant, and *p* < 0.01 according to two-way ANOVA, respectively.

AMF inoculation and DSE inoculation increased the Cd content of maize roots by 104% and 120%, respectively. Moreover, DSE inoculation significantly increased Cd accumulation in the shoots and roots of maize by 258% and 507%, respectively. According to the two-way ANOVA showed that DSE significantly increased Cd accumulation of maize. There was a very significant interaction effect between AMF and DSE on Cd content and accumulation in maize roots (**Figure 5**).

### 3.7 Correlation analysis

There were positive correlations between the ZR content and the GSH content and the activities of γ-GCS, ATPS, SIR, and OAS-TL. The IAA content was positively correlated with γ-GCS, ATPS, SIR, and OAS-TL. The ABA content was positively correlated with the GSH content and the activities of γ-GCS, GSH, ATPS, and SIR. Moreover, the ZR and IAA contents in roots positively correlated with the Tr and Pn in the leaves (**Table 2**). Thus, the endogenous hormone contents had a significant or highly significant positive correlations with GSH metabolism in maize roots and photosynthetic physiology in leaves, which plays a vital role in regulating the Cd tolerance of maize.

**Table 2:** Correlation analysis of endogenous hormone content with photosynthesis and GSH metabolism of maize.

	Pn	Tr	Gs	Ci	GSH	GR	γ-GCS	ATPS	SIR	OAS-TL
ZR	0.579^**^	0.433^*^	0.295	-0.212	0.549**	0.346	0.720**	0.559**	0.522**	0.521**
GA	0.035	0.183	0.178	-0.061	0.222	0.190	0.151	0.137	0.375	0.041
IAA	0.674^**^	0.429^*^	0.291	-0.075	0.366	0.239	0.515**	0.590**	0.519**	0.618**
ABA	0.561^**^	0.298	0.155	-0.286	0.482*	0.368	0.643**	0.501*	0.458*	0.383

“*” and “**” means *p* < 0.05 and *p* < 0.01 according to correlation analysis, respectively.

## 4 Discussion

Heavy metals inhibit plant growth and development, which can be alleviated by AMF and DSEs. AMF and DSEs can promote plant growth by improving plant photosynthesis and antioxidant physiology, and regulating plant hormone content, and enhancing host tolerance to adverse environmental conditions such as drought, cold, salinity, heavy metals, and other abiotic stresses (Ahammed et al., 2020). In the present study, inoculation with AMF and DSEs protected against Cd-induced growth inhibition and oxidative stress by regulating Cd accumulation in plants, endogenous hormone contents, and enzymes involved in GSH metabolism in maize.

Many studies have shown that AMF and DSEs regulate Cd uptake by host plants, inhibit Cd transfer from roots to leaves, and reduce the physiological toxicity of Cd to plants. For example, AMF increased the Cd concentration in the stems and roots of *Solanum nigrum* L. grown in Cd-polluted soils (Liu et al., 2015). AMF stabilizes Cd ions in soil and limits Cd absorption by plants, thus ensuring the normal metabolic function of plants, and improving the plant tolerance to Cd toxicity (Janeeshma et al., 2022). Similarly, DSE promoted the fixation of Cd in roots and limited the transfer of Cd from roots to shoots (Xiao et al., 2021). In the present study, the AMF inoculation, DSE inoculation, and AMF+DSE co-inoculation also increased the Cd content in maize roots and promoted Cd retention in the roots, which were both beneficial for enhancing plant tolerance to Cd stress. The increased Cd content in roots, which was promoted by AMF+DSE, was related to the Cd-binding capability of the fungal mycelia components, such as melanin (Ban et al., 2012). In addition, the extracellular secretion of endophytic fungi or exudates from inoculated roots and the intracellular sequestration of Cd ions are also important mechanisms for reducing Cd mobility in soil (Leonhardt et al., 2014).

Plant endogenous hormones play important roles in regulating plant growth and adaptability to abiotic stress. ZR, GA, and IAA are essential endogenous growth regulators (Gray, 2004), and ABA is a stress hormone that responds to abiotic stress (López-Climent et al., 2011). In many cases, AMF- or DSE-regulated hormones (such as ZR, GA, IAA, and ABA) contribute to improving plant growth under abiotic stress. For example, Yan and Zhong (2007) reported that AMF significantly increased the IAA content in the roots of *Cinnamomum camphora* L. and decreased the ABA content in the leaves and roots under aluminum stress. AMF increases the contents of IAA, GA, and ABA in the roots and leaves of tomato plants (He et al., 2010). Similarly, DSE colonization in roots resulted in a significant decrease in ABA levels in soybean (*Glycine max* L.) under Cu stress (Khan and Lee et al., 2013), and a decrease in ABA levels and an increase in GA levels in cucumber (*Cucumis sativus* L.) under salt stress, thereby stimulating the growth of plant roots, improving nutrient absorption, and promoting plant growth (Khan et al., 2012; Sukumar et al., 2013). The DSE significantly affected the gene expression involved in phytohormone transport and the ABA and IAA contents in maize roots, which was considered as the main reason for promoting maize growth (Wang et al., 2023). Similarly, faba beans inoculated with endophytic fungi (*Aspergillus niger* and *Penicillium chrysosporium*) promoted plant growth and nutrient uptake by regulating endogenous plant hormones under Pb stress (El-Mahdy et al., 2021). In the present study, co-inoculation of AMF+DSE significantly increased the ZR and IAA contents in maize roots and promoted maize growth, suggesting that AMF and DSE inoculation might improve host growth by coordinating the balance of hormones and improving the ability of maize to cope with Cd stress.

Furthermore, glutathione (GSH), which is a tripeptide containing sulfhydryl (-SH) groups, participates in the complexation and detoxification of Cd ions in plants (Pearson and Cowan, 2021). GSH not only plays an important role in the antioxidant process though the glutathione-ascorbic acid cycle but also directly acts as a metal-chelating agent (Guo et al., 2012). There are many enzymes involved in GSH metabolism (Bano et al., 2022). First, ATPS initiates GSH metabolism, which catalyzes sulfur assimilation, i.e., sulfate and ATP, to form adenosine phosphate sulfate (Anjum et al., 2015), and a low Cd concentration leads to an increase in ATPS activity in maize (Nazar et al., 2011). SIR is another key enzyme involved in sulfur assimilation to reduce sulfite to sulfide (Rather et al., 2020). In the last step of sulfur assimilation, OAS-TL catalyzes OAS and hydrogen sulfide to produce cysteine (Cys). Cys is the product of sulfur assimilation and the starting material for GSH synthesis. Additionally, γ-GCS is the rate-limiting enzyme for GSH synthesis, and the GSH content provides feedback to inhibit γ-GCS activity (Rennenberg et al., 2007). GR is important for GSH metabolism and can convert oxidized glutathione (GSSG) into GSH (Mishra et al., 2006). GSH and GR scavenge H_2_O_2_ (Anjum et al., 2012).

In the present study, AMF+DSE co-inoculation improved the activities of ATPS and SIR, indicating that AMF+DSE co-inoculation promoted sulfate reduction and assimilation in maize roots and met the Cys demand for GSH synthesis. In plants under Cd stress, inoculation with AMF and DSEs can enhance plant adaptability and alleviate Cd toxicity by promoting glutathione metabolism and thiol compound content in plants (Degola et al., 2015). In addition, a greater GSH content in the AMF-inoculated and DSE-inoculated plants led to feedback inhibition of γ-GCS activity, but AMF+DSE co-inoculation significantly increased γ-GCS activity in maize roots. These results indicated that GSH synthesis was enhanced by AMF and DSE, which promoted the Cd tolerance of maize. Similarly, maize inoculated with AMF (*Rhizophagus intraradices* and *Glomus versiforme*) significantly increased the GSH content, which helped to alleviate Cd toxicity and promoted maize growth (Zhang et al., 2019).

More importantly, there were positive correlations between the ZR and IAA contents and the photosynthetic physiology, the activities of γ-GCS, ATPS, SIR, and OAS-TL, and the GSH content in the present study. These correlations demonstrated the positive synergistic effects of AMF and DSE on photosynthesis, GSH metabolism, and endogenous hormone content in maize. These findings agreed with the results of Yang et al. (2015) who reported that AMF significantly enhanced the photosynthetic physiology of leaves by increasing the net Pn, Tr, and stomatal conductance. Similarly, AMF and DSEs improve photosynthesis and resistance physiology and enhance the ability of plants to resist the toxicity of heavy metals (Gadd, 2007; Ren et al., 2015).

However, several studies have shown that the co-inoculation of AMF and DSE has competitive effects on mycorrhizal colonization, maize growth, and root hydraulic conductivity under drought stress (Gong et al., 2023). Once AMF infests roots, mycelial development is inhibited by DSE fungal exudates (Scervino et al., 2009). These reports indicate the complexity of the interactions between AMF and DSEs that colonize plant roots and our lack of understanding of their working mechanisms at the physiological and molecular levels. Thus, more research on the ecological functions and interactions of AMF and DSEs is needed.

## 5 Conclusions

Under the stress of 25 mg·kg^-1^ Cd, AMF+DSE co-inoculation significantly enhanced the photosynthetic rate of maize leaves and increased the endogenous hormone contents and GSH metabolism in roots. Correlation analysis revealed that the endogenous hormone contents in maize roots was significantly related to photosynthesis and GSH metabolism. Moreover, there was a significant or highly significant positive interaction effect between AMF and DSEs on the photosynthetic rate, root GSH metabolism, and endogenous hormone content. Thus, the coexistence of AMF and DSEs had a synergistic effect on enhancing the Cd tolerance of maize.

## Data availability statement

The original contributions presented in the study are included in the article/supplementary material. Further inquiries can be directed to the corresponding authors.

## Author contributions

ZW: Formal Analysis, Writing – original draft. LW: Data curation, Formal Analysis, Writing – original draft. XL: Data curation, Formal Analysis, Writing – original draft. GZ: Investigation, Methodology, Writing – original draft. ZL: Investigation, Methodology, Writing – original draft. ZY: Writing – review & editing. FZ: Funding acquisition, Supervision, Writing – review & editing.
